# Regulation of Bcl-XL by non-canonical NF-κB in the context of CD40-induced drug resistance in CLL

**DOI:** 10.1038/s41418-020-00692-w

**Published:** 2021-01-25

**Authors:** Marco Haselager, Rachel Thijssen, Christopher West, Louise Young, Roel Van Kampen, Elaine Willmore, Simon Mackay, Arnon Kater, Eric Eldering

**Affiliations:** 1 Department of Experimental Immunology, Amsterdam UMC, University of Amsterdam, Amsterdam institute for Infection & Immunity, Cancer Center Amsterdam, Amsterdam, The Netherlands; 2 Lymphoma and Myeloma Center Amsterdam, LYMMCARE, Amsterdam, The Netherlands; 3 Department of Hematology, Amsterdam UMC, University of Amsterdam, Amsterdam institute for Infection & Immunity, Cancer Center Amsterdam, Amsterdam, The Netherlands; 4 Strathclyde Institute of Pharmacy and Biomedical Sciences, University of Strathclyde, Glasgow, UK; 5 Zuyderland Medical Center, Sittard, The Netherlands; 6 Drug Discovery Unit, Medical School, Newcastle University, Newcastle upon Tyne, UK

**Keywords:** Cancer microenvironment, Haematological diseases

## Abstract

In chronic lymphocytic leukemia (CLL), the lymph node (LN) microenvironment delivers critical survival signals by inducing the expression of anti-apoptotic Bcl-2 members Bcl-XL, Bfl-1, and Mcl-1, resulting in apoptosis blockade. We determined previously that resistance against various drugs, among which is the clinically applied BH3 mimetic venetoclax, is dominated by upregulation of the anti-apoptotic regulator Bcl-XL. Direct clinical targeting of Bcl-XL by, e.g., Navitoclax is however not desirable due to induction of thrombocytopenia. Since the actual regulation of Bcl-XL in CLL in the context of the LN microenvironment is not well elucidated, we investigated various candidate LN signals to drive Bcl-XL expression. We found a dominance for NF-κB signaling upon CD40 stimulation, which results in activation of both the canonical and non-canonical NF-κB signaling pathways. We demonstrate that expression of Bcl-XL is first induced by the canonical NF-κB pathway, and subsequently boosted and continued via non-canonical NF-κB signaling through stabilization of NIK. NF-κB subunits p65 and p52 can both bind to the Bcl-XL promoter and activate transcription upon CD40 stimulation. Moreover, canonical NF-κB signaling was correlated with Bfl-1 expression, whereas Mcl-1 in contrast, was not transcriptionally regulated by NF-κB. Finally, we applied a novel compound targeting NIK to selectively inhibit the non-canonical NF-κB pathway and showed that venetoclax-resistant CLL cells were sensitized to venetoclax. In conclusion, protective signals from the CLL microenvironment can be tipped towards apoptosis sensitivity by interfering with non-canonical NF-κB signaling.

## Introduction

One of the main problems in chronic lymphocytic leukemia (CLL) is the acquired drug resistance in response to treatment and despite the discovery and development of new targeted therapies, relapses are common. One of the hallmarks of CLL is dysregulation of apoptosis by overexpression of the anti-apoptotic protein Bcl-2, which renders CLL highly sensitive to the Bcl-2 inhibitor venetoclax (ABT-199). Venetoclax has proven very successful in clinical trials in CLL and was FDA approved for CLL with a 17p chromosomal deletion in 2016, for previously treated CLL in 2018 and for previously untreated CLL in 2019. However, a low percentage of patients reached complete remission and this may be related to microenvironment-induced resistance^1–3^.

The lymphoid microenvironment plays an important role in acquired drug resistance in CLL patients since these are the sites where CLL cells receive signals from surrounding cells that push them into a proliferative and anti-apoptotic state. Importantly, CLL cells interact with follicular T helper cells via CD40L-CD40 interactions, which provide stimuli that increase apoptosis resistance^4–8^. The anti-apoptotic proteins Bcl-XL, Bfl-1, and Mcl-1 are overexpressed in CLL cells from lymph node (LN) samples compared to peripheral blood (PB)^9^. We and others have demonstrated that CD40L stimulation of CLL cells in vitro also increased Bcl-XL, Bfl-1, and Mcl-1^3,10–12^. In addition, we have previously shown that Bcl-XL plays an important role in shifting CLL venetoclax sensitivity towards resistance in the context of CD40 stimulation^3,8^. However, direct targeting of Bcl-XL by BH3 mimetics is associated with induction of thrombocytopenia^13^. Therefore, insight into signals leading to Bcl-XL expression is required in order to assess alternative options for therapeutic targeting in CLL and other pathologies.

Upon CD40 stimulation in CLL cells and healthy B cells, NF-κB signaling is activated, correlating with the expression of Bcl-XL and Bfl-1^10,11,14^. Moreover, we have shown that LN CLL cells express higher levels of p65 and p52 compared to CLL cells isolated from PB^10^. Activation of the canonical NF-κB pathway leads to IκB kinase (IKK) phosphorylation, which in turn phosphorylates inhibitor of NF-κB (IκB)^15,16^. IκB sequesters p50/p65 dimers in the cytoplasm when NF-κB signaling is not active, but upon phosphorylation, IκB is degraded allowing for the translocation of p50/p65 dimers to the nucleus to activate transcription of target genes^15–17^. Activation of the non-canonical NF-κB pathway leads to the stabilization of NIK, which induces phosphorylation of IKKα homodimers and subsequent phosphorylation of p100^18^. Upon phosphorylation, p100 is processed into p52 which together with RelB translocates to the nucleus to activate transcription of target genes^17,19,20^. Currently, the contribution of canonical versus non-canonical NF-κB signaling in regulating Bcl-XL remains unsolved. Most studies have focused on canonical NF-κB signaling^11,21^, but our more recent data suggested that non-canonical NF-κB signaling is correlated with regulation of Bcl-XL as well^10^. In addition, it is not quite clear which external signals can contribute to or dominate Bcl-XL expression, or other pro-survival Bcl-2 proteins. Therefore, in this study we investigated the causality of canonical versus non-canonical NF-κB signaling in the expression of Bcl-XL, as well as Bfl-1 and Mcl-1. We found that non-canonical NF-kB signaling contributes strongly to Bcl-XL expression and inhibition of that route significantly blocks CD40-mediated resistance to venetoclax.

## Materials and methods

### Patient material

After written informed consent, patient blood was obtained during diagnostic or follow-up procedures at the Departments of Hematology and Pathology of the Academic Medical Center, Amsterdam. This study was approved by the AMC Ethical Review Board and conducted in agreement with the Declaration of Helsinki. Blood mononuclear cells of patients with CLL, obtained after Ficoll density gradient centrifugation (Pharmacia Biotech) were cryopreserved and stored as previously described^22^. Expression of CD5 and CD19 (BD Biosciences, San Jose, CA, USA) on leukemic cells was assessed by flow cytometry (FACS Canto; BD Biosciences). CLL samples included in this study contained 85–99% CD5^+^/CD19^+^ cells.

### Reagents

ABT-199 was purchased from Active Biochem (Bonn, Germany). A-1331852 was purchased from Chemietek (Indianapolis, IN, USA).

### Synthesis of 4-(1-(2-aminopyrimidin-4-yl)indolin-6-yl)-2-methylbut-3-yn-2-ol (CW15337)

4-(6-Bromoindolin-1-yl)pyrimidin-2-amine (73 mg, 0.25 mmol), copper iodide (9.3 mg, 0.05 mmol), and Pd(dppf)Cl_2_ (20.4 mg, 0.025 mmol) were stirred under argon in DMF (1 mL) in a sealed 0.5–2 mL microwave vial. DIPA (140 µL, 1 mmol) and 2-methylbut-3-yn-2-ol (24 µL, 0.25 mmol) were added before heating to 90 °C with stirring for 24 h. HPLC purification yielded the title product as a white solid (24 mg, 24%), LC-MS purity = 96.6%; ^1^H (DMSO-*d*_*6*_, 500 MHz) δ 1.50 (s, 1H), 3.24 (t, *J* = 7.9 Hz, 2H), 4.19 (t, *J* = 8.1 Hz, 2H), 5.42 (br s, 1H), 6.47 (d, *J* = 6.4 Hz, 1H), 7.15 (d, *J* = 7.5 Hz, 1H), 7.31 (d, *J* = 7.3 Hz, 1H), 8.05 (br s, 1H), 8.17 (br s, 2H), 8.48 (br s, 1H); ^13^C (DMSO-*d*_*6*_, 125 MHz) δ 27.15, 32.12, 49.89, 64.18, 81.23, 96.08, 98.03, 120.41, 122.04, 125.82, 128.68, 134.81, 142.23, 144.45, 155.16, 160.05; HRMS (ESI +ve) calculated for C_17_H_19_N_4_O requires 295.1553 and the found mass was 295.1556. See structural formula in Fig. 5A.

### NIK biochemical assay

Recombinant human NIK (active) 5 nM (Promega, USA) was added with myelin basic protein (0.1 mg/mL; Promega, USA) and 5 µM ATP in assay buffer (40 mM Tris-HCl, pH 7.5 ± 0.05; 20 mM MgCl_2_; 0.1 mg/mL BSA and 50 µM DTT) in a white half well 96-well plate (Greiner Bio One GmbH, Germany) in the absence and presence of CW15337 (1 nM–30 µM), to a total volume of 20 µL. The plate was covered with a lid, wrapped in cling film and placed in a foil bag and shaken on a Stuart microplate shaker (Bibby Scientific Ltd, UK) at 850 rpm for 1 min before incubation at 30 °C for 1 h. The plate was removed from foil bag and allowed to equilibrate to 21 °C for 15 min before the addition of Kinase-Glo^®^ reagent (Promega, USA). The mixture was incubated in the dark for 10 min at 21 °C and luminescence was detected on a Wallac Victor 1420 multilabel counter (Perkin Elmer, UK). IC_50_ values were converted to *K*_i_ values using the Chen–Prusoff equation.

### IKK biochemical assay

Inhibition of CW15337 against human recombinant IKKα and IKKβ was assessed using a dissociation-enhanced ligand fluorescent immunoassay as previously described^23^.

### Cell culture and detection of apoptosis

Lymphocytes of CLL patients were co-cultured with NIH3T3 fibroblasts stably transfected with human CD40L or negative control as described before^10,24^. After 24 h, CLL cells were detached by gentle resuspension and incubated with or without compounds/drugs for an additional 24 h. CLL cell viability was measured by flow cytometry using DiOC6 and TO-PRO-3 viability dyes. Specific apoptosis is defined as [% cell death in treated cells] – [% cell death in medium control] / [% viable cells medium control] × 100.

### Knockdown of Bcl-XL and NIK in primary CLL cells

CLL cells were transfected using the Amaxa nucleofection technology (Amaxa) as previously described^10^ with 3 µg siRNA targeting Bcl-XL and non-targeting siRNA (Ambion, Thermo Fisher Scientific) or 3 µg siRNA targeting NIK and non-targeting siRNA (Dharmacon). After nucleofection, cells were cultured on 3T40L for 24 h before drugs sensitivity assay was performed or protein lysates were obtained.

### Western blot analysis

Western blot analysis was performed using standard techniques^22^. Membranes were probed with the following antibodies: anti-Bcl-XL (#2764), p-p65 (#3033), and p100/p52 (#4882) (Cell Signaling, Boston, MA, USA), actin (sc-1616) (Santa Cruz Biotechnology), and anti-A1/Bfl-1 was a kind gift of Prof. Dr. J. Borst (The Netherlands Cancer Institute, Amsterdam, The Netherlands). Odyssey Imager (Li-Cor Biosciences) was used as a detection method according to the manufacturer’s protocol.

### Luciferase reporter gene experiments

The basic pGL3 luciferase reporter vector (Promega) was used to construct reporter plasmids with various lengths of the Bcl-XL and p100 promoter. The primers used for the Bcl-XL promoter were: 5′-CAGACAAAGTGCTTAACCACAAG-3′ and 5′- TTTTATAATAGGGATGGGCTCAACC-3′. The primers used for the p100 promoter were: 5′- AGAGGTTGCAGTGAGCCAAAATCC-3′ and 5′-GGTGTGGGTGCAGGCACC-3′. The plasmids pCMV4_NIK-HA and CMV4_p100 were obtained from Addgene (Cambridge, MA, USA). HEK293T cells were transfected with 1 µg of luciferase reporter plasmid with or without 0.1 µg NIK/p100 or 0.3 µg pcDNA3.1/zeo-eGFP as internal control. Polyethylenimine (Polysciences, Inc.) was used for transfection and luciferase activity was measured after 24 h by using BioTek Synergy-HT (Winooski, VT, USA). HEK293T cells were obtained from ATCC (Manassas, VA, USA), authenticated by STR profiling and tested for mycoplasma contamination.

### Real-time polymerase chain reaction

Total RNA was isolated from primary CLL cells using the GenElute Mammalian Total RNA Miniprep Kit (Sigma) and cDNA was synthesized by reverse transcriptase reactions according to the manufacturer’s instructions (Promega). Products were amplified in a Fast SYBR green (Life Technologies) reaction (40 cycles of 5 s at 95 °C followed by 30 s at 60 °C).

### Statistics

To ensure adequate power of statistical testing, sample sizes were chosen based on the type of material used, including at least two experiments in the case of cell lines and at least four patient samples in the case of primary CLL cells. Additional patient samples were included to ensure similar variation with each group of data. As for patient samples, both technical and biological replicates were included. Furthermore, patient samples were included based on IGHV mutation status when relevant. The Student’s *t*-test was used to analyze paired observations. The one-way or two-way ANOVA with multiple testing corrections were used to analyze differences between groups; *p < 0.05; **p < 0.01; ***p < 0.001; ****p < 0.0001. For every figure, statistical tests are justified as appropriate and the data met the assumptions of the tests. Finally, the variance between groups that were being statistically compared was similar.

## Results

### CD40 stimulation strongly activates NF-κB signaling and Bcl-XL expression in CLL

**Fig. 1:**
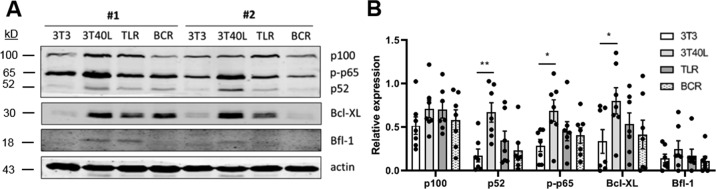
CD40 stimulation strongly activates NF-κB signaling and Bcl-XL expression in CLL. CLL cells were cultured for 24 h on fibroblasts (3T3), fibroblasts transfected with human CD40L (3T40L), stimulated with CpG, or stimulated with IL-4 and αIgM. **A** Protein lysates were probed for p100, p52, p-p65, Bcl-XL, Bfl-1, and actin as loading control. **B** Densitometric analysis of p100, p52, p-p65, Bcl-XL, and Bfl-1 are shown normalized to the actin loading control (*n* = 7). Bars represent the mean ± SEM, *p < 0.05, **p < 0.01 (two-way ANOVA).

Multiple pathways of NF-κB activation in B cells have been described, including B cell receptor (BCR) signaling, Toll-like receptor (TLR) signaling upon recognition of distinct molecular patterns, and CD40 signaling mediated by follicular T helper cells^25^. To investigate which of these signaling mechanisms dominate activation of NF-κB and Bcl-XL in CLL, we stimulated primary CLL cells with either CD40L-transfected 3T3 fibroblasts (3T40), CpG to activate TLR9, or IL-4 combined with anti-IgM to activate BCR signalling^25^ (Fig. 1A). Although we observed variation between data points, these are a result of well-known differences between CLL patients. In addition to Bcl-XL, the canonical NF-κB factor p-p65 and non-canonical p52 showed the strongest upregulation upon CD40 stimulation according to densitometric analysis (Fig. 1B). Moreover, the anti-apoptotic mediator Bfl-1, which has previously been reported to correlate with the activation of the canonical NF-κB pathway^10^, also showed the highest upregulation upon CD40 stimulation. Thus, CD40 signaling strongly activated canonical and non-canonical NF-κB signaling as well as Bcl-XL in both mutated and unmutated CLL patient samples, which led us to further investigate NF-κB-mediated drug resistance in the context of CD40 activation.

### Bcl-XL plays an important role in venetoclax drug resistance in CLL

**Fig. 2:**
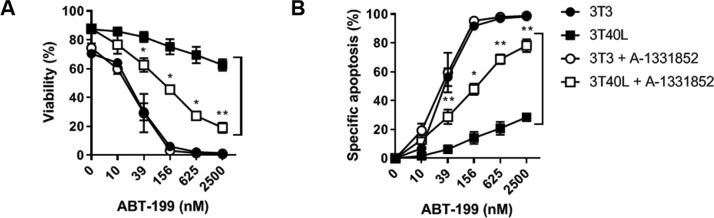
Bcl-XL plays an important role in venetoclax drug resistance in CLL. **A,B** CLL cells were cultured for 24 h on 3T3 or 3T40L. After detachment, cells were treated with a titration of ABT-199 (0–2500 nM) for 24 h with or without inhibition of Bcl-XL (A-1331852). For inhibition of Bcl-XL, a titration of 0-30-117-468-1875-7500 nM A-1331852 was used. Viability (**A**) was measured using flow cytometry using DiOC6 and TO-PRO3 viability stainings (Thermo Scientific). Specific apoptosis (**B**) was calculated. Averaged data of 4 CLL samples are shown. Bars represent the mean ± SEM, *p < 0.05, **p < 0.01 (paired *t*-test (3T40L versus 3T40L + A-1331852)).

CLL cells become resistant to a broad range of chemotherapeutic drugs after CD40 stimulation, and previous studies have demonstrated that Bcl-XL plays an important role in resistance to ABT-737 and ABT-199^3,12^. To assess the role of Bcl-XL in resistance to ABT-199, we applied BH3 mimetics specific to Bcl-XL (A-1331852) and Bcl-2 (ABT-199). The 3T40 activation of CLL cells caused significant resistance to ABT-199 compared to the 3T3 negative control due to the upregulation of Bcl-2 family members (Fig. 2A, B). When cotreated with the Bcl-XL inhibitor A-1331852, CLL cells became significantly sensitized to ABT-199, as witnessed by the shift in the response curve. These results confirmed that Bcl-XL is a major regulator of venetoclax drug resistance in CLL cells^26^.

### CD40 stimulation induces Bcl-XL expression via canonical as well as non-canonical NF-κB signaling

**Fig. 3:**
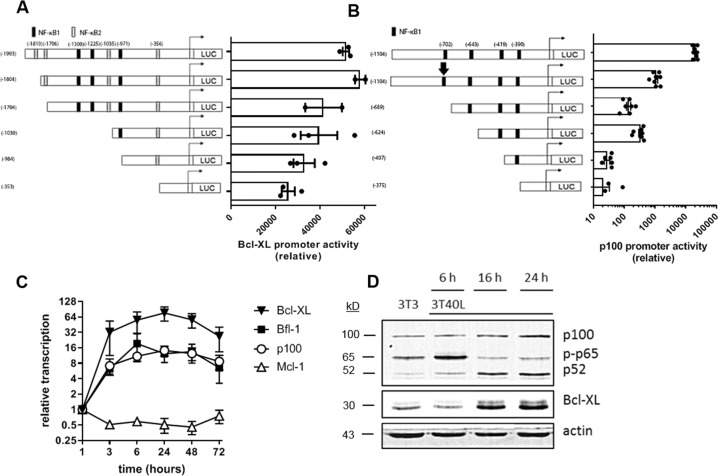
CD40 stimulation induces Bcl-XL expression via canonical as well as non-canonical NF-κB signaling. **A** HEK293T cells were transfected with luciferase reporter gene constructs carrying Bcl-XL promoter truncations. NF-κB binding sites were predicted using JASPAR database^51^. Promoter activity was measured 24 h post-transfection. Bars represent the mean ± SEM of the luciferase activation normalized to the empty pGL3-basic vector (*n* = 3). **B** HEK293T cells were transfected with luciferase reporter gene constructs carrying p100 promoter truncations, or a p100 promoter, where the first NF-κB1 binding site was scrambled (depicted by an arrow). NF-κB binding sites were predicted using JASPAR database^51^. Promoter activity was measured 24 h post-transfection. Bars represent the ± SEM of the luciferase activation normalized to the empty pGL3-basic vector (*n* = 7). **C** Bcl-XL, p100, and Mcl-1 mRNA expression by CLL cells over time after stimulation with CD40L measured by real-time PCR (*n* = 6). Results are shown as the mean ± SEM. **D** CLL cells were cultured on 3T3 or 3T40L for 6, 16, or 24 h. Protein lysates were probed for p100, p-p65, p52, Bcl-XL, and actin as loading control.

Both the canonical and non-canonical NF-κB pathways have been reported to be upregulated early after CD40 activation, correlating with the induction of Bcl-XL expression^10,11^. Potential NF-κB binding sites in the human Bcl-XL promoter consisted of three canonical and four non-canonical motifs, identified within the ~2000 bp region preceding the transcriptional start site. Since reporter assays with gene constructs carrying Bcl-XL promoter truncations are not technically feasible in primary CLL, assessing the direct role of these motifs in Bcl-XL expression was performed in HEK293T cell lines. Stepwise deletion of NF-κB binding sites gradually reduced Bcl-XL promoter activity (Fig. 3A), indicating that both canonical and non-canonical NF-κB signaling pathways play a role in activating the transcription of Bcl-XL. Crosstalk between the canonical and non-canonical NF-κB pathways has previously been reported^27,28^ and the promoter of the non-canonical NF-κB precursor p100 contains canonical NF-κB binding sites^29^. Therefore, studies with reporters carrying p100 promoter truncations were also performed in HEK293T cells. Scrambling of the first canonical binding site (depicted by an arrow) resulted in a 20-fold reduction of promoter activity, whereas deletion of this binding site reduced p100 promoter activity by 150-fold, which may be explained by structural differences (Fig. 3B). Stepwise deletion of additional binding sites further reduced p100 promoter activity. Next, CD40 stimulation of primary CLL cells induced transcription of Bcl-XL, Bfl-1, and p100 (Fig. 3C). In comparison, Mcl-1 was not upregulated at the transcriptional level. The overall timing of Bcl-XL upregulation did not appear to be different from upregulation of Bfl-1 or p100, yet the level of induction in case of Bcl-XL was greater, in correlation with the added contribution of the non-canonical pathway. At the protein level, an increase of the canonical NF-κB factor p-p65 was detected 6 h after CD40 stimulation (Fig. 3D). At 16 h post-CD40 stimulation, a decrease of p-p65 was detected as well as an increase of the non-canonical NF-κB factor p52, suggesting a shift from canonical pathway activity to the non-canonical pathway. This shift was associated with upregulation of Bcl-XL protein levels suggesting that Bcl-XL expression is caused predominantly by the activation of the non-canonical NF-κB pathway. These results indicate that the canonical NF-κB pathway is activated early after CD40 stimulation, which subsequently activates the non-canonical NF-κB pathway via p100 to further induce the expression of Bcl-XL.

### Non-canonical NF-κB signaling causes Bcl-XL expression in CLL

**Fig. 4:**
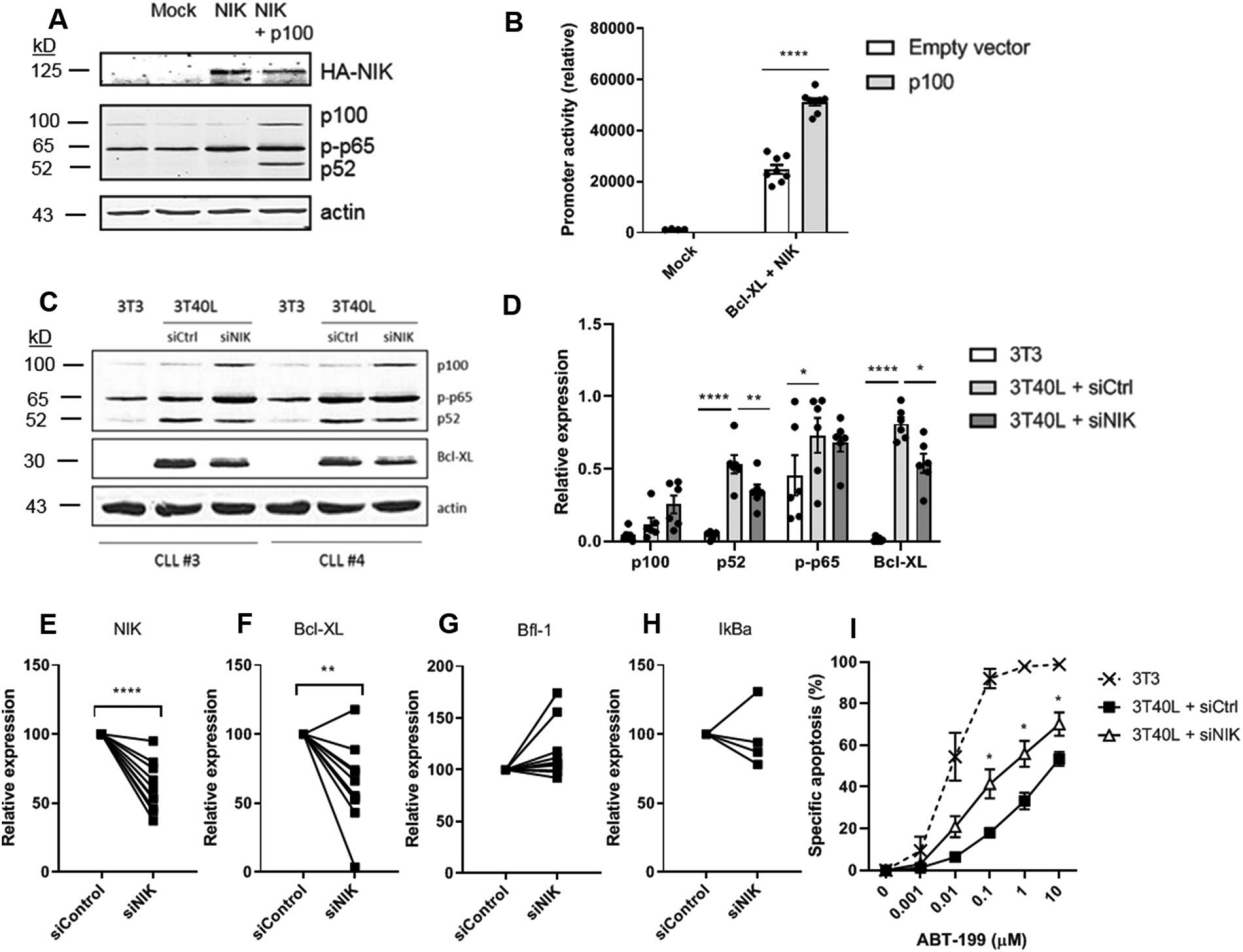
Non-canonical NF-κB signaling is critical for Bcl-XL expression in CLL. **A** HEK293T cells were transfected with empty pGL3-basic vector (mock) or HA-tagged NIK with or without cotransfection of p100. Protein lysates were probed for HA, p100, p-p65, p52, and actin as loading control. **B** HEK293T cells were transfected with Bcl-XL promoter luciferase reporter gene constructs as well as HA-tagged NIK, with or without cotransfection of p100 (*n* = 2). Promoter activity was measured and bars represent the mean ± SEM of the luciferase activation normalized to the empty pGL3-basic vector, **p < 0.01 (one-way ANOVA). **C** CLL cells were nucleofected with either a non-targeting control siRNA (siCtrl) or an siRNA targeting NIK (siNIK) and subsequently cultured on 3T40L for 24 h. After detachment, protein lysates were made and probed for p100, p-p65, p52, Bcl-XL, and actin as loading control. Blots of two representative CLL samples are shown. **D** Densitometric analysis of p100, p52, p-p65, and Bcl-XL are shown (*n* = 6). Bars represent the mean ± SEM, *p < 0.05, **p < 0.01, ****p < 0.0001 (two-way ANOVA). **E**–**H** CLL cells were nucleofected with NIK siRNA or control siRNA and cultured on 3T40L for 24 h. NIK (*n* = 10) (**E**), Bcl-XL (*n* = 10) (**F**), Bfl-1 (*n* = 10) (**G**), and IκBα (*n* = 4) (**H**) mRNA expression was measured by real-time PCR. Samples were normalized to HPRT, **p < 0.01, ****p < 0.0001 (paired sample *t*-test). **I** After detachment, cells were incubated with 0.001–10 μM ABT-199 for 24 h after which viability was measured by flow cytometry using DiOC6 and TO-PRO-3 viability dyes (*n* = 4). Bars represent the mean ± SEM, *p < 0.05 (paired sample *t*-test (3T40L + siCtrl versus 3T40L + siNIK)).

To further elucidate the role of non-canonical NF-κB signaling in Bcl-XL expression, Bcl-XL promoter activity studies were performed in HEK293T cells. HEK293T cells hardly contain endogenous p100 or NIK, thus allowing measurement of Bcl-XL promoter activity driven predominantly by active canonical NF-κB signaling, corresponding to a basal level of Bcl-XL promoter activity (Fig. 4B). When p100 and NIK were cotransfected, the processing of p100 into p52 was induced (Fig. 4A), thereby activating non-canonical NF-κB signaling, upon which Bcl-XL promoter activity was significantly increased (Fig. 4B). Additionally, transfection of NIK into HEK293T cells increased p65 phosphorylation, again indicating crosstalk between the canonical and non-canonical NF-κB signaling pathways (Fig. 4A). These data further indicate causality between non-canonical NF-κB signaling and Bcl-XL expression. To confirm this in primary CLL cells, we determined the effect of silencing NIK on Bcl-XL expression in CLL cells. At the protein level, NIK silencing inhibited the processing of p100 to p52 and significantly reduced Bcl-XL protein levels according to densitometric analysis (Fig. 4C, D). Moreover, NIK silencing significantly reduced Bcl-XL transcription in CD40L-stimulated CLL cells (Fig. 4E, F). It has previously been reported that the activation of the canonical NF-κB pathway correlates with Bfl-1 expression^10^. Therefore, we included Bfl-1 as well as the target and negative regulator of canonical NF-κB signaling IκBα, as markers for specificity of the NIK knockdown, and indeed their transcription was not significantly affected upon NIK knockdown (Fig. 4G). Finally, NIK silencing alleviated CD40-induced resistance to venetoclax (Fig. 4H). Combined, these results support that non-canonical NF-κB signaling significantly contributes to Bcl-XL expression and affects sensitivity to venetoclax in CLL.

### Targeting non-canonical NF-κB signaling downregulates Bcl-XL and increases sensitivity to venetoclax

**Fig. 5:**
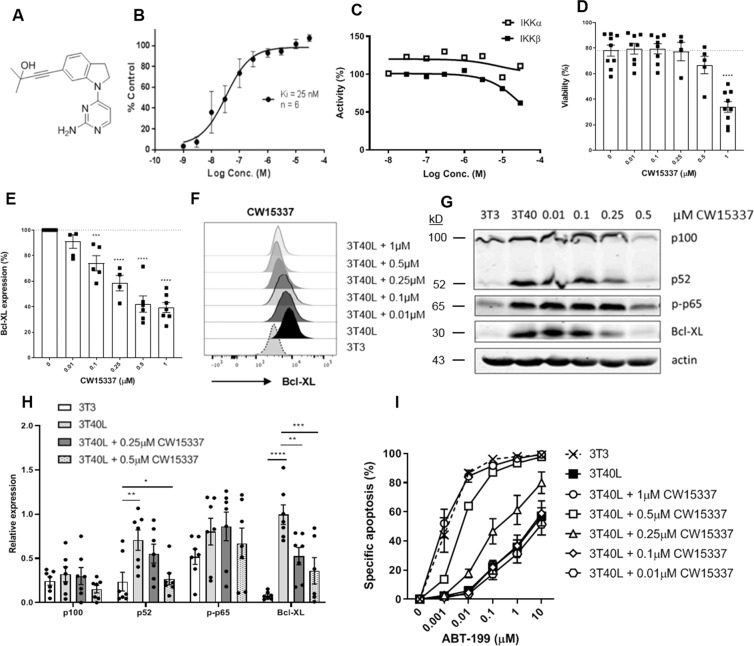
Targeting non-canonical NF-κB signaling downregulates Bcl-XL and increases sensitivity to venetoclax. **A** Structural formula of 4-(1-(2-aminopyrimidin-4-yl)indolin-6-yl)-2-methylbut-3-yn-2-ol (CW15337). **B** NIK inhibitory activity of 0.001–30 µM CW15337 was determined using the Kinase-Glo^®^ assay. Results are shown as mean ± SEM (*n* = 6). **C** IKKα and IKKβ inhibitory activity of up to 30 µM CW15337 was determined using the dissociation-enhanced ligand fluorescent immunoassay. To measure the kinase activity of IKKα/β, recombinant IKKα/β were incubated with a peptide substrate containing the phosphorylation motif of IκBα and ATP solution in presence/absence of CW15337. Kinase activity was quantified based on the amount of phosphorylated substrate relative to the untreated control. **D** CLL cells were cultured on 3T40L and simultaneously treated with a titration of CW15337 for 24 h. After detachment, viability was measured by flow cytometry using DiOC6 and TO-PRO-3 viability dyes. Bars represent the mean ± SEM (*n* = 9). **E** In addition, cells were stained intracellularly for Bcl-XL and measured by flow cytometry. Bars represent the mean ± SEM (*n* = 7). **F** MFI peaks of 1 representative sample is shown. **G** Protein lysates were probed for p100, p52, p-p65, Bcl-XL, and actin as loading control. Blot from 1 representative CLL sample is shown. **H** Densitometric analysis of p100, p52, p-p65, and Bcl-XL are shown (*n* = 7). Bars represent the mean ± SEM, *p < 0.05, **p < 0.01, ***p < 0.001, ****p < 0.0001 (two-way ANOVA). **I** After detachment, cells were incubated with a titration of 0.001–10 µM ABT-199 for 24 h after which viability was measured by flow cytometry using DiOC6 and TO-PRO-3 viability dyes. Bars represent the mean ± SEM (*n* = 8).

As part of a drug discovery program developing pharmacological agents to target the non-canonical NF-κB signaling pathway via inhibition of NIK^30^, we have been exploring the structure–activity relationship of compounds related to 4-(1-(2-aminopyrimidin-4-yl)indolin-6-yl)-2-methylbut-3-yn-2-ol (CW15337) first described in a patent filed by Amgen exploring alkynyl alcohols as kinase inhibitors^31^ (Fig. 5A). We have shown that CW15337 is a potent inhibitor of NIK (*K*_i_ = 25 nM) in a primary biochemical assay (Fig. 5B) and importantly, does not inhibit IKKα or IKKβ kinase activity up to 30 µM CW15337 since the IC50 was not reached^23^ (Fig. 5C). CW15337 therefore provides a useful chemical tool that discriminates between the canonical and non-canonical pathways through the selective targeting of NIK to further substantiate our findings. CLL cells were activated on CD40L-presenting fibroblasts and simultaneously treated with a titration of CW15337 for 24 h. Flow cytometric analysis showed low toxicity on CLL cells (Fig. 5D) with a dose–response downregulation on Bcl-XL expression (Fig. 5E, F). CD40 stimulation activated both canonical p-p65 and non-canonical p100/p52 which was associated with an upregulation of Bcl-XL (Fig. 5G). Despite some well-known variation between CLL patients, both p52 and Bcl-XL were inhibited at 0.25 µM CW15337 whereas p-p65 was unaffected, indicating specific inhibition of the non-canonical NF-κB pathway. When the dose was increased to 0.5 µM, inhibition of p-p65 was also observed. Finally, venetoclax sensitivity was tested after treatment with CW15337 (Fig. 5H). Consistent with the western blot data, abrogation of venetoclax resistance could be observed starting at 0.25 µM CW15337. Increasing the dose to 0.5 µM CW15337 almost completely sensitized CLL cells to the levels of 3T3 inactivated CLL cells, as now both the canonical and non-canonical NF-κB pathways were inhibited. These observations confirm that inhibiting the non-canonical NF-κB signaling pathway sensitizes resistant CLL cells to venetoclax by inhibiting the expression of Bcl-XL.

## Discussion

A major problem in CLL is acquired resistance in response to treatment where Bcl-XL is a key player^3,32,33^. New drugs that directly target Bcl-XL have been assessed in clinical trials for CLL^34,35^. ABT-737 was the first drug that binds and antagonizes Bcl-2 and Bcl-XL^34^. However, since blood platelets also express Bcl-XL, ABT-737 caused dose-dependent thrombocytopenia and failed in clinical trials^36,37^. Later, the newer BH3 mimetic venetoclax, which only targets Bcl-2, was introduced into the clinic. Because of Bcl-XL upregulation in LN-residing CLL cells, this may potentially contribute to reduced venetoclax sensitivity and eventual relapse. In vitro, Bcl-XL upregulation renders CLL cells highly insensitive to venetoclax^3,38^. Therefore, understanding the processes that control upregulation of Bcl-XL in response to microenvironmental stimuli is clinically relevant and may identify novel targets or approaches for therapy of CLL.

Here we investigated the regulation of Bcl-XL in the context of CD40-mediated drug resistance in CLL.

We dissected the contribution of the canonical and non-canonical NF-κB signaling pathways in the expression of Bcl-XL. A dominant role of the non-canonical signaling pathway was found and subsequently a pharmacological inhibitor of NIK was applied in order to sensitize CLL cells to venetoclax via downregulation of Bcl-XL.

The induction of Bcl-XL expression by NF-κB was already established early in B cells^11^ as well as many other cell types^21^, but the separate roles of the canonical and non-canonical NF-κB signaling pathways in the regulation of Bcl-XL had not yet been elucidated. Both canonical and non-canonical NF-κB factors can bind to the Bcl-XL promoter and contribute to Bcl-XL expression. Our data suggests that CD40-induced canonical NF-κB signaling activates target gene p100, inducing p100 protein expression which is processed into p52 as a result of phosphorylation by CD40-stabilized NIK. This suggests a model in which canonical NF-κB signaling is activated early after CD40 activation where subsequently the non-canonical pathway is activated via p100.

We showed that activity of the non-canonical pathway is associated with the upregulation of Bcl-XL in primary CLL cells. This is further supported by a significant increase in Bcl-XL promoter activity upon introduction of non-canonical NF-κB signaling in HEK293T cells using reporter activity studies. CLL patients with a deletion of NFKBIE, a negative regulator of NF-κB, show increased p65 phosphorylation and translocation, yet Bcl-XL gene expression is not significantly differently expressed^39^. This suggests that canonical NF-κB is not the sole or primary determinant of Bcl-XL expression, consistent with our data. Moreover, even when CLL cells already have high basal levels of p65, they can still be stimulated via CD40 to upregulate Bcl-XL^14^.

Conversely, Bfl-1 expression in CLL cells correlates with the activation of the canonical NF-κB pathway^10^. Knockdown of NIK resulted in a decrease of Bcl-XL transcription and protein levels which was not the case for Bfl-1. Varying degrees of Bcl-XL inhibition could be explained by the differences in expression of NIK between patient samples. Knockdown of NIK only partially alleviated resistance to venetoclax in primary CLL cells, which may be explained by the fact that the knockdown was not complete as non-canonical NF-κB signaling was still active to some degree, as observed by the presence of p52.

In contrast, we showed that Mcl-1 is not transcriptionally regulated by NF-κB upon CD40 activation, which is consistent with our previous work where we showed that CD40-induced upregulation of Mcl-1 is dependent on the PI3K-AKT-mTOR signaling pathway and independent of NF-κB activation^40^. Although several publications have reported a correlation between NF-κB activation and Mcl-1 expression in CLL in response to other stimuli^41–43^, this may possibly be due to NF-κB-induced autocrine signals mediated by interleukins which upregulate Mcl-1^42^, or a common upstream activator of Mcl-1 and NF-κB, as in the case for CD40 stimulation.

Finally, we applied a pharmacological inhibitor of NIK to further dissect the canonical and non-canonical NF-κB signaling pathways. Inhibition of the non-canonical NF-κB pathway by targeting NIK resulted in a dose-dependent downregulation of Bcl-XL in primary CLL cells. In addition, CLL cells resistant to venetoclax were sensitized again upon specific targeting of the non-canonical NF-κB pathway. At higher concentrations, the canonical pathway was also affected, as observed by reduced levels of p-p65, further abrogating resistance to venetoclax. Another compound that is structurally very similar to CW15337 has previously been reported to inhibit both canonical and non-canonical NF-κB signaling^44^. Crosstalk between NIK and p65 has been described before where it has been shown that knockout of p65 results in the accumulation of NIK^45^. In turn, high levels of NIK trigger the accumulation of p65^46^. Furthermore, a direct role for NIK in canonical NF-κB signaling was shown based on the interaction of NIK with IKKα^47^. Consistently, an identified homozygous 17q21 mutation in MAP3K14 impaired the activity of NIK resulting in defective non-canonical and canonical NF-κB signaling^48^. The observation that NIK is involved in both canonical and non-canonical NF-κB pathways in the context of lymphomas has also been confirmed by in vitro studies^49^.

ABT-737 represented a novel mechanism of drug-induced thrombocytopenia via direct targeting of Bcl-XL. Therefore, indirect targeting of Bcl-XL via regulatory signaling presents an alternative potential therapeutic strategy. Unable to synthesize more Bcl-XL, platelets are primed for cell death as the Bcl-XL inherited from megakaryocytes degrades over time, thereby determining platelet life span^37^. Further studies are required to elucidate whether targeting Bcl-XL via inhibition of NIK, as reported here for CLL, would also affect Bcl-XL in platelets. Although it is unclear how Bcl-XL is regulated in megakaryocytes, Bcl-XL mutant mice showed increased platelet clearance yet normal megakaryocytopoiesis. Importantly, adoptive transfer experiments showed that platelet clearance was independent of the recipient’s genotype, meaning that platelets from healthy donors exhibited a normal half-life. Therefore, potential thrombocytopenia following indirect Bcl-XL inhibition can be addressed by platelet transfusion which is not the case for ABT-737 administration. Another study using transgenic mice showed that overexpression of Bcl-XL in megakaryocytes lead to impaired platelet fragmentation, whereas platelet number was not significantly affected^50^. This suggests that platelet homeostasis is tightly regulated and that upon indirect Bcl-XL inhibition, megakaryocytes will fragment earlier into platelets, thereby preventing a significant change in platelet levels.

In summary, we have identified Bcl-XL as an important regulator of CLL drug resistance, we have shown that Bcl-XL is predominantly regulated via non-canonical NF-κB signaling and that interfering with this signaling pathway can tip the balance of resistant CLL cells towards apoptosis sensitivity.

## Supplementary information

Supplementary Figure Legends

Additional Western blot data included in the quantification shown in Figure 1B.

Additional Western blot data included in the quantification shown in Figure 4D.

Additional Western blot data included in the quantification shown in Figure 5H.
