# Supplementary material for: Regulation of Bcl-XL by non-canonical NF-κB in the context of CD40-induced drug resistance in CLL
Source: Cell Death Differ. 2021 Jan 25;28(5):1658–68. doi: 10.1038/s41418-020-00692-w (PMC8167103; doi:10.1038/s41418-020-00692-w)
Supplement: Supplementary file 1 — Supplementary Figure Legends [file 41418_2020_692_MOESM1_ESM.doc]

**Supplementary** **Figure Legends**

**Supplementary Table 1. Patient table.** Overview of patient characteristics of primary CLL samples included in this study, including cell lines.

**Supplementary Figure 1. Additional Western blot data included in the quantification shown in Figure 1B.** CLL cells were cultured for 24 hours on fibroblasts (3T3), fibroblasts transfected with human CD40L (3T40L), stimulated with CpG, or stimulated with IL-4 and αIgM. Protein lysates were probed for p100, p52, p-p65, Bcl-XL, Bfl-1 and actin as loading control.

**Supplementary Figure 2. Additional Western blot data included in the quantification shown in Figure 4D.** CLL cells were nucleofected with either a non-targeting control siRNA (siCtrl) or an siRNA targeting NIK (siNIK) and subsequently cultured on 3T40L for 24 hours. After detachment, protein lysates were made and probed for p100, p-p65, p52, Bcl-XL and actin as loading control.

**Supplementary Figure 3. Additional Western blot data included in the quantification shown in Figure 5H.** CLL cells were cultured on 3T40L and simultaneously treated with a titration of CW15337 for 24 hours. After detachment, protein lysates were probed for p100, p52, p-p65, Bcl-XL and actin as loading control. Bcl-XL at 0.5µM CW15337 in CLL #16 was excluded in the densitometric analysis due to an artefact.
